# Navigating Knowledge Landscapes in the digital society: a look back and visions for the future

**DOI:** 10.3325/cmj.2018.59.274

**Published:** 2018-10

**Authors:** Anna Lydia Svalastog, Joachim Allgaier, Srećko Gajović

**Affiliations:** 1 Østfold University College, Department of Health and Social Studies, Fredrikstad, Norway; 2 RWTH Aachen University, HumTec – Human Technology Center, Aachen, Germany; 3 University of Zagreb School of Medicine, Croatian Institute for Brain Research, Zagreb, Croatia *srecko.gajovic@hiim.hr*

## Why did we establish the Navigating Knowledge Landscapes Research Network?

**Figure 1:**
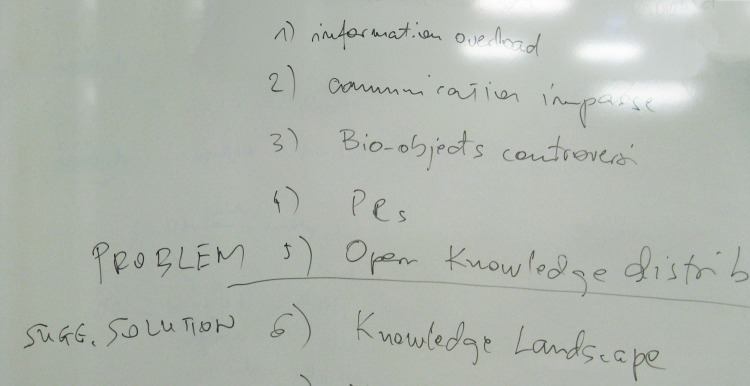
Whiteboard from the Lecture Room at the Department of Histology and Embryology, University of Zagreb School of Medicine, where Srećko Gajović has just written “knowledge landscape” as a suggested solution to the above problems. Since those present at the meeting in Zagreb on January 14, 2014 (Joachim Allgaier, Srećko Gajović, Lucia Martinelli, and Anna Lydia Svalastog) appreciated this particular term, Anna Lydia Svalastog made a photo for the record.

The digital society has altered global communications, relations, and organization of various public services. New challenges and opportunities presented by the digital society make it necessary to find ways of describing and discussing the present situation. This need created the background for establishing the Navigating Knowledge Landscapes (NKL) Network. The term “Knowledge Landscapes,” representing 3D knowledge distribution in both off- and online arenas, was coined at the Zagreb meeting of the Task Force on Communication of the COST Action 1001 Bio-Objects and Their Boundaries, Governing Matters at the Intersection of Society, Politics and Science in January 2014 (Figure 1). This led to the first publication using the term “Knowledge Landscapes” in the *Croatian Medical Journal* (*CMJ*) (1). Subsequently, the three authors of this text bravely decided that they would continue and expand our interdisciplinary collaboration on Knowledge Landscapes to explore how innovative knowledge is communicated and distributed in the digital society. The decision was made in Brussels on December 4, 2014, after the Final Conference of the Bio-objects COST Action.

## Short history

During 2014, Allgaier and Svalastog, with Østfold University College as a co-applicant, received funding from Austrian Alpen-Adria-Universität Klagenfurt, to further strengthen the collaboration. This funding ensured Allgaier’s visit to Svalastog at Østfold University College in Norway in February 2015. The visit made it possible to develop the first draft of Allgaier’s and Svalastog’s text on the Ebola outbreak (2). It also provided space and time for discussions, which were presented to Gajović remotely via Skype. At this point, we also started to extend the group, and the network quickly began to grow, leading to the first NKL Network meeting in Zagreb in September 2015. Gajović, who at the time was the Editor-in-Chief of the *CMJ*, invited the new NKL Network to contribute with a new publication stream as a successor of the Bio-objects stream. As the network grew steadily we found this opportunity very interesting and decided to go for it. We put our thoughts into texts, and by August 2015 we had published an editorial in the *CMJ* presenting the goals to be achieved by the NKL Network (3). This resulted in a series of 18 NKL-texts published in the *CMJ* as the Network’s main publishing platform.

During the first three years, the NKL Network became a visible agent in the European debate on health in the digital society. There was a NKL-session at the DIPEx (Database of Individual Patients’ Experiences) International Conference on Narratives of Health and Illness at La Laguna, Tenerife, on November 11-12, 2016. DIPEx International is an association of expert researchers qualitatively investigating people’s personal experiences of health and illness (4). In the aftermath of the conference a formal collaboration between DIPEx International and NKL Network was established. A formal collaboration was also set up between the Society for Risk Analysis Europe and the NKL Network. Finally, at the Lošinj Days of Bioethics at Mali Lošinj, Croatia, in 2017 and 2018, we connected the topic of Knowledge Landscapes with the topics from integrative bioethics.

The NKL Network was envisioned as a basis for further collaboration and development of joint research activities. To develop a new international research network was fun, but also demanding. We would not have managed without the funding from Østfold University College and the Norwegian Research Council. The funding ensured regular meetings between Svalastog and Gajović, and launching of the first NKL International Conference in Halden on April 27, 2017 and the second in Zagreb in December 2018.

## Collection of texts published in the Croatian Medical Journal

The texts published in the *CMJ* constitute a body of thinking about challenges that concern health in the digital society. These texts can be organized in four parts.

### Navigating health and science communication – ambitions and goals

These were the first two texts to present the intentions and key concepts of our work. Within the frame of the Bio-objects COST Action, we had already coined the concept of Knowledge Landscapes (2). In the Editorial in August 2015 we focused on the act of navigation through Knowledge Landscapes (1).

### The digital society – altered conditions for/and health

One area of interest was health in the digital society. Among the changes introduced by the digital society was the online overload of information. Digital availability of “everything” creates a syncretism of ideas and high levels of complexity (5). For the individuals who search for answers on the internet, the answers will vary depending on the filters used for browsing and “personalizing” the search. The concept of the “filter bubble,” which describes the result of the work of the filtering algorithms, is important for understanding how health (mis-)information functions (6). It also implies that the knowledge landscapes that occur are tentative (7). Similar to the filter bubble is the concept of echo chamber, and both have become widely-used terms, pointing toward the user as the reference of the outcome (8). As the online interactions can represent a movement, influence the way of thinking, or form a social group, ie, something that is framing the individual, black hole is suggested as a slightly different metaphor than filter bubble or echo chamber. The black hole implies societal forces that attract participants and take over the control of the participants’ discussions (9). In black holes, the outcomes are predefined, and the users are trapped in the self-reinforcing environment used to persuade and distribute an opinion.

Due to innovations and new technologies, human beings are no longer the only inhabitants of the knowledge landscapes, as the digital mediators (ie, digital entities with human-like features) are crawling through the landscapes. They interact with humans, produce the contents, and influence opinions, subsequently altering the access to the knowledge relevant for health (10).

### In a different knowledge landscape – situated but not lost

In addition to understanding the new conditions established by the digital society, we reflected on its implication on the individual, both privately and professionally. For example, in a clinical situation, how does one achieve patient autonomy? As the patients are now part of the digital society, the health professional can consider and discuss the data stemming from the patient’s own search for information, and help patients interpret their meaning (11). As an extension of the individual who is the subject of a medical intervention, there are digital health records, biological properties, etc. Questions that concern the ethical regulation and usage of digital health records, health databases, and bio-banks are heterogeneous and integrated. Presenting navigation across knowledge landscapes could be more timely and fruitful than analyses anchored in the domain in which a database is located, be it clinical, scientific, or commercial (12). Together with adults, children are also present in the digital environment, which presents new challenges for social workers and the society as a whole. What do we know about children’s online life and how do we understand and approach it (13)? Alternatively, for patients with chronic diseases, the internet might be used not only to make sense of a symptom or condition, but as a source of knowledge in the creation of a meaningful life-story. In this process it might be important for health practitioners to acknowledge the patient’s search for new knowledge as an effort to create meaning (14). The online discourses regarding health are complex, exemplified by the Croatian discourses on the conscientious objections of physicians to conduct abortion. In the Croatian case, the discourse is complex on several levels, for example in regard to the participants in the discourse vs actual stake holders (there is a lack of health professionals in the debate) (15). The online discourse is also complex due to a blurred or missing relation between the stakeholder’s understanding of the situation and actual research-based knowledge. Thus, the centers of gravity that shape the knowledge landscape rarely represent scientific or medical community. With its flexible geography, the digital society creates new knowledge landscapes that produce opportunities for the patients as citizens to govern their own properties and gain new knowledge. This can relate to the basic postulates of the health care and societal justice (16).

### The plurality of cultures in the digital society

The pluralities of cultures are fundamental to understanding knowledge and, therefore, very important for the NKL Network. They refer to the plurality of meaning, which is something other than anachronisms and disagreements. The cultural implications of the digital society do not seem to undermine or limit cultural plurality, although they need to be comprehended to avoid conflict and misunderstandings. In situations of a pandemic like the Ebola outbreak it was crucial that all the involved parties understand and respect each other’s cultures. It related to how and why action was understood as threatening, and how and why conspiracy theories were quickly developed and how discontinuity in communication was resolved (2). Technological innovations, for example in the field of reproductive technology, may represent a transition into a new cultural era. So, employers who make egg freezing possible for their female workers to postpone childbirth apply technical solution to a social problem. In this sense, the development mirrors how working arena and motherhood still represent conflicting interests in the modern society (17). The example from Indigenous people in Australia shows how Aboriginal methods and knowledge systems are considered and sought after online, representing the use of social media and mobile phones as a tool for knowledge transfer between two separate knowledge landscapes, official and indigenous (18). The digital society can facilitate renewed ways of fulfilling individuals’ health needs and strengthen community ties. Video ethnographic methods capture interactions, discourse, performance, narratives, body language, culture, law, and ceremony, and are shared via social media and mobile phones in a culturally relevant manner, making the new technology an essential component of the health promotion tool kit of the future. Due to the complexity of contemporary knowledge landscapes, communication is crucial. To navigate these conversations, for example, between law, science, and bioethics, implies navigating traditions/cultures/symbolic systems and understandings and definitions of a phenomenon different in fundamental ways, like the meaning of the embryo as a controversial entity (19). The digital society is a widespread phenomenon, and yet another question is how to understand children’s agency in the digital realm (20). One might also say that the multicultural character of globalized modern societies – eg, in academic life, public debates, or international events – is made visible or even put on display in highly networked digital societies.

## Looking forward

The key theme for NKL-network has been health in the digital society. Our approach has been broad and included somatic, mental, and social health and well-being. Our strong belief was that to be in charge of one’s own health, we need to empower the citizen, and the key to achieve it is trustworthy knowledge and communication. The question, however, is how to get trustworthy and reliable information without being overwhelmed by the many offers of the digital society. An important move toward trustworthy information and trustworthy dissemination consists of investigating what information is distributed and how, and how it affects individual’s decisions. Looking forward, we will need different types of professionals to be informed about how the digital society affects the individuals they work with (21). In addition, the professionals need to be able to discuss the digital society with those who are concerned, and the employers need to include the activities involving the digital environment into job descriptions. We will need more detailed research on the various knowledge landscapes established by the digital society for different individuals and professionals, as well as case studies on different groups’ and professionals’ individual navigations in these landscapes, and the relations to their specific needs. We will also need new methodologies (eg, various variants of data mining) to describe and analyze the wider picture of internet contents and different behaviors on the internet.

The new conditions existing in the digital society include the online information overload, complex multi-directed communications, and extreme social potency. In addition, the accumulation and distribution of knowledge, by experts and lay people, are interwoven with economic relations, legal, and administrative regulations. Looking forward, we will need new forms of risk analyses. We need concepts and theories describing how the digital society works and in what ways power unfolds in these new information ecosystems. And we need to develop strategies on how to make necessary health-related knowledge accessible to the citizens. This also includes information on how to handle health-related situations in daily life.

When reflecting on previous NKL-texts, it is obvious that the digital society is not simply good or bad, but that it primary represents a challenge. We need to investigate and better understand how it is different from earlier times. As such, the theories of modernity, even late-modernity or post-modernity, need to be critically reviewed and revised. The examples used in the NKL texts point toward a strengthened global-local relation and to complex situations regarding the navigation in knowledge landscapes in the context of cultural plurality.

NKL Network's success depends on international and interdisciplinary collaborations, and its members' fruitful contribution to the exploration of online and offline communication, as well as of distribution of medical information and its impact on treatment, recovery, well-being, and quality of life.

## References

[1] Svalastog AL, Allgaier J, Martinelli L, Gajovic S (2014). Distortion, confusion, and impasses: could a public dialogue within knowledge landscapes contribute to better communication and understanding of innovative knowledge?. Croat Med J.

[2] Allgaier J, Svalastog AL (2015). The communication aspects of the Ebola virus disease outbreak in Western Africa–do we need to counter one, two, or many epidemics.. Croat Med J.

[3] Svalastog AL, Allgaier J, Gajović S (2015). Navigating knowledge landscapes: on health, science, communication, media, and society.. Croat Med J.

[4] DIPEx International. Avilable from: http://www.dipexinternational.org/About-us/. Accessed: October, 7, 2018.

[5] Svalastog AL, Donev D, Kristoffersen NJ, Gajović S (2017). Concepts and definitions of health and health-related values in the knowledge landscapes of the digital society.. Croat Med J.

[6] Pariser E. The filter bubble: What the Internet is hiding from you. London: Penguin UK; 2011.

[7] Holone H (2016). The filter bubble and its effect on online personal health information.. Croat Med J.

[8] Barberá P, Jost JT, Nagler J, Tucker JA, Bonneau R (2015). Tweeting from left to right: Is online political communication more than an echo chamber?. Psychol Sci.

[9] Gajović S, Svalastog AL (2016). When communicating health-related knowledge, beware of the black holes of the knowledge landscapes geography.. Croat Med J.

[10] Šimunić D, Kerner A, Gajović S (2018). Digital mediators as key enablers of navigation towards health in knowledge landscapes.. Croat Med J.

[11] Ringstad O (2016). Patient autonomy in a digitalized world: supporting patients’ autonomous choice.. Croat Med J.

[12] Aicardi C, Del Savio L, Dove ES, Lucivero F, Tempini N, Prainsack B (2016). Emerging ethical issues regarding digital health data. On the World Medical Association Draft Declaration on Ethical Considerations Regarding Health Databases and Biobanks.. Croat Med J.

[13] Hansen HA, Bjorktomta SB, Svalastog AL (2017). Digital society generates new challenges on child welfare services.. Croat Med J.

[14] Ringstad 0 (2016). Being an autonomous person with chronic disease.. Croat Med J.

[15] Borovečki A, Babić-Bosanac S (2017). Discourse, ethics, public health, abortion, and conscientious objection in Croatia.. Croat Med J.

[16] Gajovic S (2018). Knowledge-for-data trade at the interface between precision medicine and person centered care.. Croat Med J.

[17] Martinelli L, Busatta L (2015). Galvagni, Piciocchi C. Social egg freezing: a reproductive chance or smoke and mirrors?. Croat Med J.

[18] Kariippanon K, Senior K (2018). Re-thinking knowledge landscapes in the context of Grounded Aboriginal Theory and online health communication.. Croat Med J.

[19] Piciocchi C, Martinelli L (2016). The charge of definitions in a multidisciplinary landscape: the case of human embryo and pre-embryo identification.. Croat Med J.

[20] Björktomta SB, Hansen HA (2018). Child welfare services and social media – childhood, being and becoming in a digital society.. Croat Med J.

[21] Toreld EM Haugli KO Svalastog AL Maintaining normality when serving a sentence in the digital society. Croat Med JForthcoming 10.3325/cmj.2018.59.335 PMC6330768 30610776

